# Evaluation of Allostatic Load as a Marker of Chronic Stress in Children and the Importance of Excess Weight

**DOI:** 10.3389/fped.2019.00335

**Published:** 2019-08-07

**Authors:** Valeria Calcaterra, Federica Vinci, Giulia Casari, Gloria Pelizzo, Annalisa de Silvestri, Mara De Amici, Riccardo Albertini, Corrado Regalbuto, Chiara Montalbano, Daniela Larizza, Hellas Cena

**Affiliations:** ^1^Pediatric Unit, Department of the Mother and Child Health, Fondazione IRCCS Policlinico San Matteo and Department of Internal Medicine University of Pavia, Pavia, Italy; ^2^Pediatric Surgery Department, Children's Hospital “G. di Cristina”, ARNAS “Civico-Di Cristina-Benfratelli”, Palermo, Italy; ^3^Biometry & Clinical Epidemiology, Scientific Direction, Fondazione IRCCS Policlinico San Matteo, Pavia, Italy; ^4^Immuno-Allergy Laboratory, Pediatric Clinic, Fondazione IRCCS Policlinico San Matteo, Pavia, Italy; ^5^Laboratory of Clinical Chemistry, Fondazione IRCCS Policlinico San Matteo, Pavia, Italy; ^6^Laboratory of Dietetics and Clinical Nutrition, Department of Public Health, Experimental and Forensic Medicine, University of Pavia, Pavia, Italy; ^7^Clinical Nutrition and Dietetics Service, Unit of Internal Medicine and Endocrinology, ICS Maugeri IRCCS, Pavia, Italy

**Keywords:** allostatic load, cumulative biological dysregulation, stress, excess weight, obesity, children

## Abstract

**Introduction:** Allostatic load (AL) refers to the physiological response associated with the burden of chronic stress. Excessive weight is an important source of physiological stress that promotes a detrimental chronic low-inflammation state. In order to define a correlation between cumulative biological dysregulation and excess weight, we measured AL scores in a pediatric population.

**Patients and Methods:** We enrolled 164 children and adolescents (11.89 ± 3.89). According to their body mass index (BMI) threshold, subjects were classified as normal in the BMI < 75th percentile, overweight in the BMI 75–95th percentile or obese in the BMI >95th percentile. Data based on 16 biomarkers were used to create the AL score. A dichotomous outcome for high AL was defined in those who had more than four dysregulated components.

**Results:** High AL was noted in 88/164 subjects (53.65%), without significant differences between genders (*p* = 0.07) or pubertal status (*p* = 0.10). Subjects with a high AL, in addition to a higher BMI (*p* < 0.001), showed higher WC and WC/HtR (*p* < 0.001), triglycerides (*p* = 0.002), fasting blood glucose (*p* = 0.03), insulin resistance (*p* < 0.001), systolic (*p* < 0.001) and diastolic blood pressure (*p* = 0.001), GGT (*p* = 0.01), PCR (*p* = 0.01), and calprotectin (*p* < 0.01) as well as lower HDL cholesterol (*p* = 0.002) than subjects with a low AL. The rate of the cumulative biological dysregulation increased progressively with increases in BMI (*p* < 0.001).

**Conclusions:** A high AL was associated with excess weight. AL may be considered a significant factor correlated with increased morbidity in children who are overweight/obese.

## Introduction

Stress is a complex process involving individual resources, vulnerabilities as well as the environment (1). Psychological, behavioral and physiological adaptations to survival requirements are necessary for an individual's capacity to adapt (2–4). Chronic exposure to stressful stimuli, is referred to as *allostatic load* (AL), and results in “wear and tear” of the adaptive regulatory systems resulting in biological alterations that weaken stress adaptive processes and increase disease susceptibility (2, 3). Chronic dietary imbalances, for example as observed in western diets rich in fats and refined sugars that lead to excessive weight gain, may affect physiological performance promoting chronic low-inflammation that is detrimental for both the physical and mental status (5–7). The consequential increase in pro-inflammatory cytokines, such as IL-6, leads to increased hypothalamic-pituitary-adrenal (HPA-) axis activity (8) and promotes chronic stress. In this study, we measured AL scores in a Caucasian pediatric population, in order to define a correlation between cumulative biological dysregulation and excess weight.

## Patients and Methods

### Patients

We consecutively enrolled 164 Caucasian children and adolescents (79 females and 85 males) aged 11.89 ± 3.89 y (range 2.6–18.0 y). The subjects were referred to our institution for auxological evaluation or obesity by their general practitioner or by their primary care pediatric consultant, between October 2017 and March 2018.

After receiving information on the nature of the study, written informed consent was obtained from the parents of the patients or the participants. Participants were excluded from the study if they had concurrent chronic or acute illnesses, any known secondary syndromes with or without obesity, or were on any medications. According to body mass index (BMI) cutoffs and age-sex percentiles (9) subjects were classified as normal for weight: BMI < 75th percentile, overweight: BMI 75–95th percentile or obese: BMI >95th percentile.

### Methods

#### Allostatic Load Definition

We used 16 biomarkers to create an AL summary score. In the original definition of the AL index, (2) biomarkers of cardiovascular, metabolic, and endocrine stress regulatory systems were included. Acknowledging the important role that inflammation plays in the response to stress and many diseases, inflammation biomarkers were added at a later time (3). As previously reported (10), since the AL biomarker panel is not univocal (11), we included markers for cardiovascular, metabolic, inflammatory and stress hormone status (11). As markers of cardiovascular and metabolic activity, in addition to BMI, we included parameters that are usually considered in metabolic syndromes, such as, waist circumference (WC), waist to height ratio (WHtR), blood pressure, fasting lipid and blood glucose levels, insulin resistance, transaminases and homocysteine levels. The latter is recognized as an independent risk factor for cardiovascular disease (12). As inflammatory markers we included calprotectin (13), IL-6 and PCR; while the serum cortisol level was measured as a marker of stress.

As previously described (10) for each biomarker, a dichotomous variable was constructed in which 0 and 1, respectively, correlated with values under the clinical cutoffs for age and gender and those in the pathological clinical range. Risk factors were summed to calculate a total AL score and the population median for the sum of the dysregulated components in our sample was 4. All patients who scored <4 were considered low AL and vice versa for all the others. As previously mentioned (10) we used this dichotomous scoring system to achieve a well-matched sample size and consequently obtained more statistical power.

#### Auxological Parameter Evaluation

In all patients, weight, height, waist circumference (WC), pubertal stage according to Marshall and Tanner (14, 15) and blood pressure were measured. BMI and the waist-to-height ratio (W/HtR) were also calculated. Weight, height, BMI, waist circumference and blood pressure were measured as previously reported (13). A BMI cut-off above the 75th percentile, indicative of an overweight status or obesity, was considered pathological; WC was considered pathological when >95th percentile for age and sex (16). Abdominal obesity was defined with a W/HtR >0.5 (17). Hypertension was defined for SBP or DBP as equal to or above the 95th percentile for age and sex (18).

#### Metabolic, Hormonal, and Inflammatory Parameters

Metabolic and hormonal parameters included fasting blood glucose (FBG), total cholesterol, high-density lipoprotein (HDL) cholesterol; triglycerides (TG), aspartate aminotransferase (AST), alanine aminotransferase (ALT), gamma-glutamyl-transferase (GGT), homocysteine, insulin and cortisol levels. The formula: insulin resistance = (fasting plasma insulin in mUI/L × fasting plasma glucose in mmol/L/22.5) was used to calculate the homeostasis model assessment for insulin resistance (HOMA-IR).

Elevated FBG was defined with values exceeding 100 mg/dl and impaired insulin sensitivity (ISI) with an HOMA-IR exceeding the 97.5th percentile for age and sex (19). Lipid fasting levels were considered pathological for total-cholesterol (TC) and TG values >95th percentile and an HDL cholesterol level <5th percentile for age and sex (20). Pathological ALT (normal value 11–39 mU/ml) and AST (normal value 11–34 mU/ml) and/or GGT (normal value 11–53 mU/ml) levels identified subjects with abnormal hepatic function.

Fasting blood glucose, insulin, total cholesterol, HDL, TG, AST, ALT, GGT, and PCR were measured with clinical chemistry methods using the Advia XPT (Siemens Healthcare). Morning serum cortisol was measured using the chemiluminescent enzyme immunoassay Immulite (Siemens Healthcare; intra- and inter-assay precision were 5.2 and 6.1%, respectively). Plasma homocysteine levels were measured with a competitive immunoassay using direct, chemiluminescent technology (Advia Centaur, Siemens Healthcare; intra- and inter-assay precision were 3.7 and 4.7%, respectively). Serum IL-6 was titered using an enzyme-linked immunosorbent assay kit (R and D Systems Minneapolis; range 0–12.5 pg/ml; intra- and inter-assay precision were 2.6 and 4.5%, respectively). Serum calprotectin was measured with a commercial ELISA assay (intra- and inter-assay precision were 8.0 and 9.0%, respectively) as previously described (13).

### Statistical Analysis

The Shapiro-Wilk test was used to determine normal distributions of quantitative variables and the results are reported as the mean and standard deviation (SD). Gender and pubertal stages were considered as qualitative variables and summarized as counts and percentages and compared between groups with the chi-square test. Associations between dichotomous AL (high vs. low) and clinical, metabolic and hormonal data were evaluated by the Student *t*-test for independent samples. The non-parametric test for trends across ordered groups was used to assess the effect of obesity on cumulative biological dysregulation. All tests were two-sided and a *p*-value < 0.05 was considered statistically significant. The STATA statistical package was used to perform data analyses (release 15 January 2017, Stata Corporation, College Station, Texas, USA).

## Results

Based on the BMI percentile threshold, 49 of the 164 (29.88%) enrolled patients were normal for weight and 115 (70.12%) were overweight or obese. Tables 1, 2 show the biomarker distributions utilized for the AL summary score, and the number of dysregulated values used to generate the score.

**Table 1:** Parameter distributions used to create the allostatic load (AL) summary score.

**Parameters**	**Absent**	**Present**
Body mass index > 75th percentile	49 (29.88%)	115 (70.12%)
Waist circumference > 95th percentile	57 (34.76%)	107(65.24%)
Waist to height ratio > 0.5	68 (41.46%)	96 (58.54%)
Systolic blood pressure ≥ 95th percentile	144 (87.8%)	20 (12.2%)
Diastolic blood pressure > 95th percentile	153 (93.29%)	11 (6.71%)
High density lipoprotein (HDL) cholesterol < 5th percentile	113 (81.09%)	31 (18.91%)
Total cholesterol > 95th percentile	149 (90.85%)	15 (9.15%)
Triglycerides > 95th percentile	152 (92.68%)	12 (7.32%)
Fasting blood glucose > 100 mg/dl	161 (98.17%)	3 (1.83%)
HOMA-IR > 95th percentile	136 (82.92)	28 (17.18%)
Pathological ALT (nv 11–39 mU/ml) and AST (nv 11–34 mU/ml) and/or GGT (nv 11–53 mU/ml)	162 (98.78%)	2 (1.22%)
Homocysteine (nv 5–13.9 μmol /L)	132 (80.48%)	32 (19.52%)
PCR > 0.5 mg/L	145 (88.41%)	19 (11.59%)
Pathological IL-6 (nv 0–12.5 pg/ml)	160 (97.56%)	4 (2.44%)
Pathological calprotectin (nv 0.3–1.6 μg/ml)	75 (45.73%)	89 (54.27%)
Elevated morning cortisol level (nv 4.3–22.4 μg /ml)	163 (99.39%)	1 (0.61%)

*nv, normal value; HOMA-IR, homeostasis model assessment for insulin resistance*.

**Table 2:** Number of dysregulated components used to create the allostatic load summary score.

**Number of AL parameters**	**Percentage of patients (%)**	**Cumulative percentage of patients (%)**
0	14.02	14.02
1	12.80	26.83
2	4.27	31.10
3	15.24	46.34
4	15.24	61.59
5	17.68	79.27
6	11.59	90.85
7	5.49	96.34
8	1.22	97.56
9	1.83	99.39
10	0.61	100

A high AL score was observed in 88/164 subjects (53.65%), without significant differences between genders (*p* = 0.07) or pubertal status (*p* = 0.10). In Table 3, we report the correlations between AL, clinical data and metabolic/hormonal parameters. Subjects with a significantly higher BMI (*p* < 0.001), WC and WC/HtR (*p* < 0.001), triglycerides (*p* = 0.002), fasting blood glucose (*p* = 0.03), GGT (*p* = 0.01), PCR (*p* = 0.01), calprotectin (*p* < 0.01) insulin resistance (*p* < 0.001), systolic (*p* < 0.0019), and diastolic blood pressure (*p* = 0.001) values as well as lower HDL cholesterol (*p* = 0.002) levels had a higher AL score.

**Table 3:** Correlation between allostatic load (AL), clinical data, and biochemical/hormonal parameters.

**Parameters**	**Low AL (<4)**	**High AL (≥4)**	***p*_value**
Height (cm)	146.59 ± 22.82	150.58 ± 15.47	0.18
Weight (Kg)	45.22 ± 17.02	59.92 ± 17.65	<0.001
Body Mass Index (kg/m^2^)	21.23 ± 11.88	25.8 ± 3.88	<0.001
Waist circumference (cm)	72.47 ± 10.78	84.98 ± 9.71	<0.001
Waist to height ratio > 0.5	0.34 ± 0.04	0.55 ± 0.52	<0.001
Pubertal status^*^			
Tanner stage 1	30 (39.47)	25 (28.41)	0.10
Tanner stages 2–3	40 (52.63)	55 (62.50)	
Tanner stages 4–5	6 (7.80)	8 (9.09)	
Systolic blood pressure (mmHg)	106.54 ± 9.84	112.82 ± 10.15	<0.001
Diastolic blood pressure (mmHg)	66.53 ± 8.26	70.57 ± 8.04	0.001
High density lipoprotein cholesterol (mg/dL)	55.73 ± 11.32	49.61 ± 11.02	0.002
Total cholesterol (mg/dL)	152.87 ± 22.95	160.17 ± 29.55	0.14
Triglycerides (mg/dL)	60.04 ± 29.2	80.98 ± 41.8	0.002
Fasting blood glucose (mg/dL)	72.89 ± 10.57	76.71 ± 2.04	0.03
HOMA-IR	1.46 ± 1.13	2.57 ± 1.96	<0.001
Aspartate Aminotransferase (mU/ml)	23.08 ± 5.45	23.33 ± 13.26	0.9
Alanine Aminotransferase (mU/ml)	14.84 ± 5.36	17.56 ± 9.16	0.06
Gamma-Glutamyl Transferase (mU/ml)	11.83 ± 4.64	14.58 ± 6.34	0.01
Homocysteine (μmol /L)	12.68 ± 7.52	13.11 ± 3.14	0.67
PCR (mg/L)^**^	0.04 (0.02–0.1)	0.16 (0.03–0.48)	0.01
IL-6 (pg/ml)^**^	1.97 (0.8–5.7)	2.83 (1.51–5.77)	0.23
Calprotectin (μg/ml)^**^	1.1 (0.8–1.9)	2.6 (1.7–4.3)	<0.001
Cortisol (μg/ml)	8.20 ± 3.97	8.85 ± 4.89	0.5

*AL, allostatic load*.

* *Data are expressed as the number of patients (percentage)*.

** *Data are expressed as the median (interquartile range)*.

A significant correlation between high AL score and excess weight was observed (87/88 of patients with pathological BMI had a high AL score vs. 1/49 in normal weight subjects *p* < 0.001). It was also noted that an increasing BMI correlated with increased cumulative biological dysregulation (*p* < 0.001).

## Discussion

In our pediatric population, we observed a significant correlation between high AL and overweight/obesity status and an altered cardio-metabolic profile. The cumulative biological dysregulation rose in the higher BMI categories. AL may be considered a significant factor correlated with increased morbidity in overweight/obese children.

Cumulative biological system dysregulation has been closely linked to AL (21) and has been shown to be associated with a poor health status, thus representing a key physiological impairment factor in the prodromal stages of disease (22). We detected a correlation between high AL and overlapping metabolic syndrome (MetS) parameters, such as abdominal obesity, high triglyceride levels, insulin resistance and hypertension, confirming our previous results showing a significant MetS increase with increased obesity severity in children (23)^.^ Therefore, these results suggest that AL may forecast important clinical consequences including cardiovascular events and metabolic disease, as well as increased mortality (2, 3).

In the children and adolescents, high AL scores correlated with excess weight and increases in the BMI categories. As reported by Foss et al. (8), obesity represents a disruption of homeostasis and may be considered a potential physiological stress inducer. While it seems evident that stress induces obesity, the reverse is not well-documented. Our data support that this hypothesis should be seriously considered at the pediatric age, where stress experience is limited compared to data reported on adults. Chronic low-grade activation of the inflammatory system is a common feature of obesity and is associated with increased secretion of pro-inflammatory cytokine, such as IL-1, IL-6 and tumor necrosis factor, that trigger stimulation of HPA axis by the adipocytes, thereby increasing cortisol release in order to limit the inflammation reaction (24).

Another feature of obesity is increased leptin secretion (25, 26) that appears to be functionally connected with the HPA axis in humans (27, 28). Hyperleptinemia, which is commonly observed in obesity, can also play a role in the stress reaction.

In our population, we also detected a correlation between high AL and calprotectin levels, which has previously been associated with obesity-related chronic low-grade inflammation in children (13). The role of this molecule on the risk of developing comorbid inflammatory conditions, should be considered.

Finally, food quality and the amount of energy consumed in relation to daily recommended requirements are key determinants of the effects that contribute to chronic disease. Dietary patterns have been associated with higher AL scores (29), indicating the negative impact of refined and simple sugars as well as saturated and trans fatty acids in long term causes of chronic degenerative diseases (30, 31).

We acknowledge that the study has humble limitations starting from the relatively small sample size, which could have limited the analysis. Therefore, future studies with larger cohorts are mandatory. Additionally, even though our observational study revealed a higher cumulative biological dysregulation in patients with excessive weight increases, this does not prove causality and it is difficult to determine whether AL is a cause or consequence of obesity. In this pediatric population, longitudinal studies would be useful to define the impact of cumulative biological dysregulation on health. Finally, we evaluated a cohort of children in which several stressors may have simultaneously affected AL scores, including unhealthy lifestyle, chronic low-grade inflammation and hormonal dysregulation. Further research aimed at single stress parameter analysis is necessary to confirm the relationships between excess weight and cumulative biological dysregulation.

In conclusion, we describe a correlation between cumulative biological dysregulation and excess weight in children and adolescents, suggesting that AL could be a useful index of health risk in the pediatric population offering a valuable measure to predict complications in obese children and promote early tailored interventions to enhance their quality of life.

## Data Availability

The raw data supporting the conclusions of this manuscript will be made available by the authors, without undue reservation, to any qualified researcher.

## Ethics Statement

This study was approved by the Institutional Review Board of the Fondazione IRCCS Policlinico San Matteo, Pavia (Protocol number: 20150005231). After receiving information on the nature of the study, written informed consent was obtained from the patients' parents or the patients themselves.

## Author Contributions

VC, DL, and HC designed the experiments, provided patient samples, wrote, and supervised the manuscript. GP wrote and supervised the manuscript. FV, GC, CR, and CM designed the experiments and provided the patient samples. AdS performed the statistical analysis. MD and RA performed the biochemical analysis. All authors approved the final version of the manuscript.

### Conflict of Interest Statement

The authors declare that the research was conducted in the absence of any commercial or financial relationships that could be construed as a potential conflict of interest.

## References

[1] Ottino-González J Jurado MA García-García I Segura B Marqués-Iturria I Sender-Palacios MJ. Allostatic load is linked to cortical thickness changes depending on body-weight status. Front Hum Neurosci. (2017) 11:639. 10.3389/fnhum.2017.00639 29375342 PMC5770747

[2] Seeman M Stein Merkin S Karlamangla A Koretz B Seeman T. Social status and biological dysregulation: the “status syndrome” and allostatic load. Soc Sci Med. (2014) 118:143–51. 10.1016/j.socscimed.2014.08.002 25112569 PMC4167677

[3] Seeman TE McEwen BS Rowe JW Singer BH. Allostatic load as a marker of cumulative biological risk: MacArthur studies of successful aging. Proc Natl Acad Sci USA. (2001) 98:4770–5. 10.1073/pnas.081072698 11287659 PMC31909

[4] McEwen BS Bowles NP Gray JD Hill MN Hunter RG Karatsoreos IN. Mechanisms of stress in the brain. Nat Neurosci. (2015) 18:1353–63. 10.1038/nn.4086 26404710 PMC4933289

[5] Castanon N Lasselin J Capuron L. Neuropsychiatric comorbidity in obesity: role of inflammatory processes. Front Endocrinol. (2014) 5:74. 10.3389/fendo.2014.00074 24860551 PMC4030152

[6] Guillemot-Legris O Muccioli GG. Obesity-induced neuroinflammation: beyond the hypothalamus. Trends Neurosci. (2017) 40:9–17. 10.1016/j.tins.2017.02.005 28318543

[7] Nguyen JCD Killcross AS Jenkins TA. Obesity and cognitive decline: role of inflammation and vascular changes. Front Neurosci. (2014) 8:375. 10.3389/fnins.2014.00375 25477778 PMC4237034

[8] Foss B Dyrstad SM Stress in obesity: cause or consequence? Med Hypotheses. (2011) 77:7–10. 10.1016/j.mehy.2011.03.011 21444159

[9] Cacciari E Milani S Balsamo A Spada E Bona G Cavallo L Italian cross-sectional growth charts for height, weight and BMI (2–20 years). J Endocrinol Invest. (2006) 29:581–93. 10.1007/BF03344156 16957405

[10] Calcaterra V Cena H de Silvestri A Albertini R De Amici M Valenza M Pelizzo G. Stress measured by allostatic load in neurologically impaired children: the importance of nutritional status. Horm Res Paediatr. (2017) 88:224–30. 10.1159/000477906 28693012

[11] Juster RP McEwen BS Lupien SJ. Allostatic load biomarkers of chronic stress and impact on health and cognition. Neurosci Biobehav Rev. (2010) 35:2–16. 10.1016/j.neubiorev.2009.10.002 19822172

[12] Ganguly P Alam SF. Role of homocysteine in the development of cardiovascular disease. Nutr J. (2015) 14:6. 10.1186/1475-2891-14-6 25577237 PMC4326479

[13] Calcaterra V De Amici M Leonard MM De Silvestri A Pelizzo G Buttari N. Serum calprotectin level in children: marker of obesity and its metabolic complications. Ann Nutr Metab. (2018) 73:177–83. 10.1159/000492579 30189427

[14] Marshall WA Tanner JM Variations in patterns of pubertal changes in boys. Arch Dis Child. (1969) 45:13–23. 10.1136/adc.45.239.13 PMC2020414 5440182

[15] Marshall WA Tanner JM Variations in patterns of pubertal changes in girls. Arch Dis Child. (1969) 44:291–303. 10.1136/adc.44.235.291 5785179 PMC2020314

[16] McCarthy HD Jarrett KV Crawley HF. The development of waist circumference percentiles in British children aged 5.0–16.9 y. Eur J Clin Nutr. (2001) 55:902–7. 10.1038/sj.ejcn.1601240 11593353

[17] Maffeis C Banzato C Talamini G Obesity Study Group of the Italian Society of Pediatric Endocrinology and Diabetology. Waist-to-height ratio, a useful index to identify high metabolic risk in overweight children. J Pediatr. (2008) 152:207–13. 10.1016/j.jpeds.2007.09.021 18206690

[18] National High Blood Pressure Education Program Working Group on High Blood Pressure in Children and Adolescents The fourth report on the diagnosis, evaluation, and treatment of high blood pressure in children and adolescents. Pediatrics. (2004) 114:555–76. 10.1542/peds.114.2.S2.555 15286277

[19] d'Annunzio G Vanelli M Pistorio A Minuto N Bergamino L Iafusco D. Insulin resistance and secretion indexes in healthy Italian children and adolescents: a multicentre study. Acta Biomed. (2009) 80:21–8. 19705616

[20] Expert Panel on Detection Evaluation and Treatment of High Blood Cholesterol in Adults. Third report of the National Cholesterol Education Program Expert Panel on Detection, Evaluation and Treatment of High Blood Cholesterol in adults (Adult Treatment Panel III). Bethesda: National Heart, Lung, and Blood Institute (2001).

[21] Beckie TM. A systematic review of allostatic load, health, and health disparities. Biol Res Nurs. (2012) 14:311–46. 10.1177/1099800412455688 23007870

[22] Lee KH Park SW Ye SM Kim SY Kim SY Han JS. Relationships between dietary habits and allostatic load index in metabolic syndrome patients. Korean J Fam Med. (2013) 34:334–46. 10.4082/kjfm.2013.34.5.334 24106586 PMC3791341

[23] Calcaterra V Klersy C Muratori T Telli S Caramagna C Scaglia F Cisternino M Larizza D. Prevalence of metabolic syndrome (MS) in children and adolescents with varying degrees of obesity. Clin Endocrinol. (2008) 68:868–72. 10.1111/j.1365-2265.2007.03115.x 17980007

[24] Mastorakos G Ilias I. Interleukin-6: a cytokine and/or a major modulator of the response to somatic stress. Ann N Y Acad Sci. (2006) 1088:373–81. 10.1196/annals.1366.021 17192581

[25] Kelly AS Metzig AM Schwarzenberg SJ Norris AL Fox CK Steinberger J. Hyperleptinemia and hypoadiponectinemia in extreme pediatric obesity. Metab Syndr Relat Disord. (2012) 10:123–7. 10.1089/met.2011.0086 22217186 PMC3339383

[26] Farr OM Gavrieli A Mantzoros CS. Leptin applications in 2015: what have we learned about leptin and obesity? Curr Opin Endocrinol Diabetes Obes. (2015) 22:353–9. 10.1097/MED.0000000000000184 26313897 PMC4610373

[27] Malendowicz LK Rucinski M Belloni AS Ziolkowska A Nussdorfer GG. Leptin and the regulation of the hypothalamic-pituitary-adrenal axis. Int Rev Cytol. (2007) 263:63–102. 10.1016/S0074-7696(07)63002-2 17725965

[28] Nye EJ Bornstein SR Grice JE Tauchnitz R Hockings GI Strakosch CR. Interactions between the stimulated hypothalamic-pituitary-adrenal axis and leptin in humans. J Neuroendocrinol. (2000) 12:141–5. 10.1046/j.1365-2826.2000.00431.x 10718909

[29] Van Draanen J Prelip M Upchurch DM. Consumption of fast food, sugar-sweetened beverages, artificially-sweetened beverages and allostatic load among young adults. Prev Med Rep. (2017) 10:212–17. 10.1016/j.pmedr.2017.11.004 29868371 PMC5984206

[30] Mattei J Noel SE Tucker KL. A meat, processed meat, and French fries dietary pattern is associated with high allostatic load in Puerto Rican older adults. J Am Diet Assoc. (2011) 111: 65. 1498–506. 10.1016/j.jada.2011.07.006 21963016 PMC3185297

[31] Mattei J Demissie S Falcon LM Ordovas JM Tucker K. Allostatic load is associated with chronic conditions in the Boston Puerto Rican Health Study. Soc Sci Med. (2010) 70:1988–96. 10.1016/j.socscimed.2010.02.024 20381934 PMC2907654

